# Pathways of Cross-Modal Access to the Visual Cortex in Late Blindness

**DOI:** 10.21203/rs.3.rs-8196909/v1

**Published:** 2026-01-20

**Authors:** Samuel Paré, James Isaac Lubell, Sylvain Baillet, Ron Kupers, Maurice Ptito

**Affiliations:** University of Montreal; Aarhus University; McGill University; University of Montreal; University of Montreal

**Keywords:** cross-modal plasticity, functional connectivity, MEG, audition, touch

## Abstract

This study investigated how auditory and tactile inputs reach the visual cortex in late-blind (LB) adults, a population for whom the neural mechanisms supporting cross-modal plasticity remain incompletely understood. Using magnetoencephalography (MEG), we measured neural responses to tactile finger stimulation and preceding auditory cues in LB and sighted controls (SC). When analyses were time-locked to the tactile stimulus, LB individuals showed markedly earlier and stronger activations in primary visual cortex (V1) than SC, with responses emerging at 35 ms versus 59 ms, suggesting the recruitment of a fast, likely monosynaptic thalamo-cortical, pathway in LB but not in SC. In contrast, analyses time-locked to the auditory cue revealed a common sequence in both groups: activation of the thalamus (~ 20 ms), followed by primary auditory cortex (A1, 35–38 ms), and then V1 (49–53 ms), consistent with direct A1-to-V1 transmission. Connectivity analyses showed enhanced thalamus-to-V1 alpha-band connectivity in LB for both auditory and tactile inputs, and increased beta-band connectivity only in the auditory modality. These effects did not depend on duration or onset age of blindness. Overall, the findings indicate that auditory and tactile signals reach V1 through distinct but complementary pathways in late blindness, highlighting substantial cross-modal reorganization in adulthood.

## Introduction

It is now well established that the brain repurposes its visual cortex for cross-modal sensory processing in individuals blind from birth. The reorganized areas participate in the processing of non-visual sensory inputs, including tactile, auditory, and olfactory information [1–4]. To achieve such extensive reorganization, the brain must establish routes whereby non-visual inputs gain access to the (cortical) visual system. Two main mechanisms have been proposed for this rerouting. First, many studies have suggested that the brain strengthens pre-existing cortico-cortical connections, thereby redirecting non-visual signals to the visual cortices in order to support efficient cross-modal processing [5–8]. Second, another line of evidence has indicated that congenital or very early blindness may result in atypical thalamic projections that re-route non-visual information to the occipital cortex [5]. The latter possibility aligns generally with established neurodevelopmental processes entailing early overproduction of synapses followed by activity-dependent pruning [9–12]. In the visual system, competition among retinal inputs normally drives this pruning process, with lesser elimination of the more active connections [13]. In the absence of retinal input at birth, however, the lacking competition permits non-visual modalities (tactile, auditory, and olfactory) to dominate in the visual system. Consequently, connections that would typically be eliminated may persist into adulthood, providing a substrate for atypical thalamo-cortical pathways in congenitally blind (CB) individuals [5]. Consequently, in CB individuals, tactile inputs can be transmitted to the primary visual cortex (V1) through this non-canonical thalamo-cortical route considerably faster reaching V1 as early as 35 ms after stimulus onset compared to the longer latency required for signals to reach the occipital cortex via the existing polysynaptic cortico-cortical pathway involving at least four synapses [14].

In contrast, in late-onset blindness (LB) the brain must adapt to vision loss either during the concluding phase of developmental maturation or later, after anatomical and functional networks are already firmly established. The connectome of LB individuals has not been characterized to the same extent as that of the CB, a state of affairs calling for clarification of the degree of plasticity in LB. Attaining such knowledge would help determine critical time windows for intervention, particularly as the incidence of LB rises with increased longevity and in association with age-related diseases [15, 16]. These considerations underscore the need for further development of vision restoration prostheses, sensory substitution devices, and optimized rehabilitation strategies [4].

In the present study, we presented electro-tactile stimuli to the fingers, which were preceded by an auditory cue, in order to examine the pathways by which tactile and auditory signals reach the visual cortex in LB individuals. The first analysis focused on tactile processing, testing the hypothesis that tactile signals reach the visual cortex via a fast thalamo-cortical pathway, consistent with prior findings in CB individuals. The second analysis extended that investigation to auditory processing, testing the hypothesis that activity in the visual cortex in LB is driven by a rapid connection between primary auditory (A1) and visual (V1) cortices as reported in CB individuals [17]. Response latencies in tactile, auditory, and visual regions were measured using magnetoencephalography (MEG), which provides millisecond-level temporal resolution.

## Methods

### Participants and ethics

Eleven LB participants (4 females; 58.0 ± 7.05 y.) and eleven age- and sex-matched sighted controls (SC; 4 females; 57.8 ± 11.1 yrs) were included in the study. In the LB group, the mean age at blindness onset was 25.7 ± 11.3 years (range: 7–41 yrs), and the mean duration of blindness was 32.0 ± 12.9 yrs (range: 8–50 yrs). The mean blindness duration index (BDI) calculated as in [18]:

BDI=(currentage–ageatblindnessonset)/currentage

was 0.55 ± 0.22 (range: 0.19–0.88), indicating that, on average, LB participants had been blind for approximately half of their lives. All LB participants were completely blind and without residual vision. Participants from both groups were blindfolded during the MEG measurements. None of the participants had any recent history of neurological or psychiatric disorders to report, nor were they taking illicit drugs or psychotropic medications at the time of imaging. In accordance with the Declaration of Helsinki, all participants gave their written informed consent to take part in study, which had approval from the University of Copenhagen local ethics committee.

All methods were performed in accordance with the relevant guidelines and regulations, and adhered to the principles of the Declaration of Helsinki. Participants gave their written informed consent and the study was approval by the University of Copenhagen local ethics committee. Demographic characteristics of the LB group are summarized in Table 1.

### Experimental Paradigm

We used the experimental paradigm as described by Müller et al. 2019 [5], which consists of a simple tactile reaction time task with four attentional conditions based on auditory cues. Participants were instructed to orient their attention toward the side of an initial auditory cue and then respond as quickly as possible to subsequent tactile stimulations by pressing a button using the hand receiving the stimulus. Tactile stimuli were presented 1000 ms after the auditory cue. The experiment comprised four conditions designed to manipulate the congruence between auditory and tactile inputs. In the attended normal condition, monaural auditory cues were congruent with the side of subsequent tactile stimulation. In the unattended normal condition, the auditory cues were monaural but incongruent with the side of tactile stimulation. The cue-only condition consisted of monaural auditory cues that were not followed by tactile stimulation, thereby creating an expectation violation. Finally, in the no-trial cue condition, a binaural auditory cue was presented to indicate that no tactile stimulation would follow.

Each participant underwent 720 trials, consisting of 640 normal trials (480 attended and 160 unattended), 40 cue-only trials, and 40 no-trial cue trials in randomized sequence (see Table 2). The experiment was divided into 10 blocks of 72 trials each, with each session lasting approximately 6 minutes and separated by a 2-minute break. Only the data from the normal trials are discussed in this paper.

### Stimulations

The auditory cues used were 50 ms lasting simple tones (trigger delay: 14 ms) produced by a custom-designed sound generator (KAR Audio, Finland) located outside the shielded room and conveyed to the participants via plastic tubes connected to ear tips. The triggers were sent via the parallel port to another PC used for data recording and sampled simultaneously with the MEG recordings. The electrotactile stimulations were administered through bipolar ring electrodes on the left and right index fingers with two stimulus current generators (SGC V3.0, SN, DeMeTec, Germany) to generate single electric square-wave pulses (200 μs). Before conducting the MEG experiments, the detection threshold (THR) for each subject was assessed using an adaptive staircase method consisting of a 1-step-up, 2-step-down procedure with seven reversals, requiring a minimum of 30 trials. This established the minimal stimulus intensity detectable by participants 70% of the time. Subsequently, we determined the comfort limit (CL) using a 3-step-up, 1-step-down procedure, also with seven reversals and a minimum of 30 trials, thereby identifying the intensity perceived as distinct but non-painful 80% of the time. Following protocols from previous studies involving electrocutaneous stimulation, we then calculated each participant’s supra-threshold (SUPRA) intensity as SUPRA = CL + (0, 2 × r), where r = CL – THR. The staircase and stimulation procedures were programmed in PsychoPy, an open-source software for psychophysics. Execution of the scripts controlled the timing of events (sounds and electrical stimuli) and the unique trigger associated with each event. As noted above, triggers were sent via the parallel port to another computer used for data recording and were sampled concurrently with the MEG recordings.

### Magnetic Resonance Imaging (MRI)

Structural MRI scans were acquired for co-registration and spatial analysis of the MEG data. T1-weighted images with 1-mm isotropic voxels were obtained using a 3D magnetization-prepared rapid gradient echo (MPRAGE) sequence on a 3 Tesla (3T) MRI system. The scans were performed on either a Siemens Tim Trio scanner (TR = 2420 ms, TE = 3.7 ms, flip angle = 9°, inversion time = 960 ms) or a Siemens Skyra scanner (TR = 2300 ms, TE = 3.8 ms, flip angle = 8°, inversion time = 938 ms) (Siemens Medical Systems, Erlangen, Germany).

### Magnetoencephalography (MEG)

MEG data were collected at the Center for Functionally Integrated Neuroscience (CFIN), Aarhus University Hospital, using a 306-channel Elekta Neuromag TRIUX system. The system consisted of 102 sensor triplets, each comprising two orthogonal planar gradiometers and one magnetometer, with a sampling rate of 1 kHz and an online band-pass filter of 0.10–330 Hz. Data acquisition took place in a magnetically shielded room equipped with Elekta’s active flux compensation system (MaxShield). At the start of each session, head position was determined using four head-position indicator (HPI) coils, which were affixed to the forehead (n = 2) and mastoid areas (n = 2). Anatomical landmarks the left and right preauricular points (LPA, RPA) and the nasion were digitized with a Polhemus Fasttrack system. An empty-room recording was performed prior to participant measurements, and these recordings were processed identically to the experimental data.

### Data Analyses

**Figure 1.**
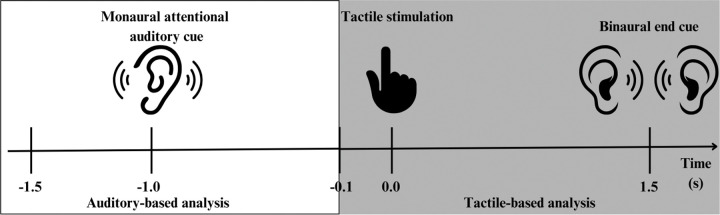
Schematic representation of the two analyses. Normal trials are presented. The white panel illustrates the auditory-based MEG analyses. The grey panel illustrates the tactile-based MEG analyses.

The MEG recordings were obtained from the single experimental paradigm described previously that presented participants with auditory cues and subsequent tactile stimulation. To test our hypotheses, we implemented two analyses that differed only in their temporal alignment. Specifically, the first analysis was time-locked to the onset of tactile stimulation, whereas the second was time-locked to the onset of the auditory cue (Fig. 1). Both analyses were performed using quasi-identical preprocessing, source reconstruction, and statistical procedures, which are described in detail in the following sections.

MEG data preprocessing began with environmental noise removal using MaxFilter software with default parameters [19]. Harmonics and 50 Hz power line artifacts were attenuated with notch filters, while cardiac and ocular artifacts were corrected using Signal-Space Projection (SSP) in Brainstorm [20]. Head position within the MEG helmet was adjusted for each run using digitized points.

Source reconstruction was performed using individual MRI volumes. Forward and inverse models were computed with a multi-sphere analytical head model and a weighted minimum-norm estimate (wMNE) with unconstrained source orientations. Brainstorm’s recommended parameters were applied: depth-weighting order = 0.5, and noise-covariance regularization = 0.1. Cortical surfaces were segmented with FreeSurfer [21]. In both analyses, regions of interest (ROIs) were defined using the Brodmann atlas. In the tactile stimulus-based analysis, the ROIs consisted of the primary somatosensory cortex (S1), V1 and a third ROI, corresponding to the posterior thalamus, which was manually segmented in Montreal Neurological Institute (MNI) space. In the auditory cue-based analysis, the ROIs consisted of the primary auditory cortex (A1), primary visual cortex (V1) and the manually segmented posterior thalamus.

Group-level comparisons involved three complementary approaches. First, we applied a z-score wMNE distributed model to the MEG source time series, providing an estimate of current strength and orientation across ROIs and the whole cortex. Second, response latencies were extracted using a multiple linear regression (MLR) model of the ROI contributions, as described by Coffey et al. [22] and Müller et al. 2019 [5]. This model reduces MEG sensor data to a weighted linear combination of the absolute forward fields of each ROI, thereby rectifying both the data and forward fields to capture ROI contributions independently of current direction. Regression coefficients over time were then used to quantify the ROI’s influence, offering high spatial specificity and sensitivity even for deep structures such as the thalamus [23], although computationally limited to three or four ROIs. Third, we assessed effective connectivity with phase transfer entropy (PTE), quantifying the directed connectivities in the alpha (8–12 Hz), beta (12–30 Hz), and gamma (40–80 Hz) bands across ROIs for every trial [24]. For the wMNE model, individual source data were projected onto a spatially smoothed Colin27 brain template [25]. For the tactile-base analysis, source time series from ~ 120 averaged trials (epoch: −1750 to 500 ms relative to tactile stimulation) were baseline corrected (−1750 to −1500 ms) and transformed into z-scores. For the auditory-based analysis, an average of 300 trials per participant (–500 to + 900 ms relative to the auditory cue) were baseline corrected (−500 to −100 ms) and converted into z-scores. In both analyses, group differences across the cortex were assessed with non-parametric permutation t-tests [26] and used primarily as descriptive, hypothesis-guided maps complementing the ROI-based analyses.

For the MLR model, ROI forward fields were extracted and normalized using a min–max scaling procedure to account for depth and size effects [27]. The normalized topographies were entered as regressors in trial-wise regressions across each epoch. In the tactile-based analysis, we modelled contralateral ROIs (thalamus, S1 and V1) relative to the electrotactile stimulus. In the second analysis, we modelled both ipsilateral and contralateral ROIs (thalamus, A1 and V1) relative to the auditory cue onset. Time series of beta-coefficients were scaled between 0 and 1, averaged across homologous contralateral ROIs, and subjected to a second min–max normalization across time. Finally, PTE analyses were computed on all trials (epoch: −100 to 500 ms), and group-level differences were assessed using Wilcoxon rank-sum tests with Bonferroni correction across ROIs and frequency bands, as required due to the non-normal distribution of the data.

## Results

### Somatosensory processing

#### Behavioral Performances

**Figure 2.**
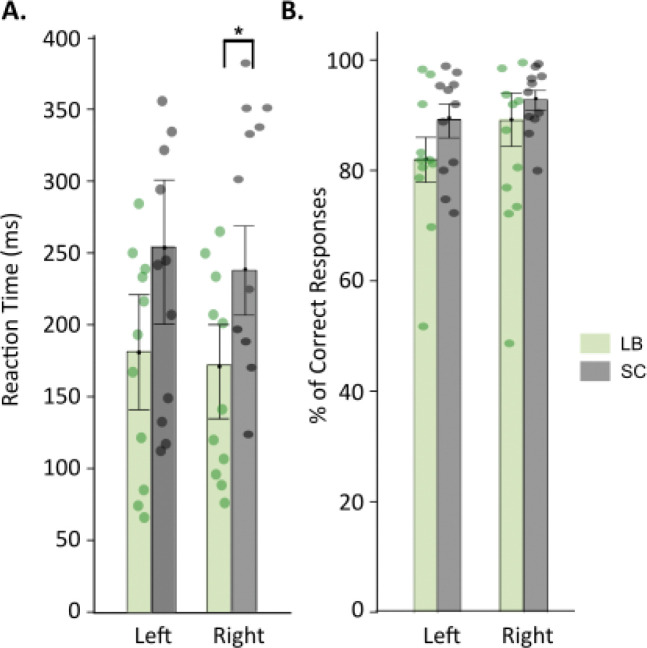
Group comparison of reaction times (A) and percentage correct responses (B) in the tactile detection task. Significant results at p < 0.05 are highlighted by an asterisk (*). LB data are shown in green whereas SC data are shown in grey. LB: late blind individuals; SC: sighted controls.

Reaction time analyses (Fig. 2A) revealed that LB participants responded significantly faster than SC when their right hand was stimulated (LB: 176 ± 38 ms; SC: 253 ± 34 ms; t(20) = −3.91, p = 0.03, unpaired *t*-test, Bonferroni corrected). A similar trend was observed for left-hand stimulation (LB: 168 ± 39 ms; SC: 229 ± 51 ms), although this difference did not reach statistical significance (*p* = 0.07, unpaired *t*-test). There were no significant group differences in response accuracy (Fig. 1B), either for left-hand stimulation (LB: 81 ± 6%; SC: 88 ± 5%); *p* = 0.12, unpaired *t*-test) or right-hand stimulation (LB: 86 ± 7%; SC: 92 ± 4%; *p* = 0.19, unpaired *t*-test).

#### Imaging Results

**Figure 3.**
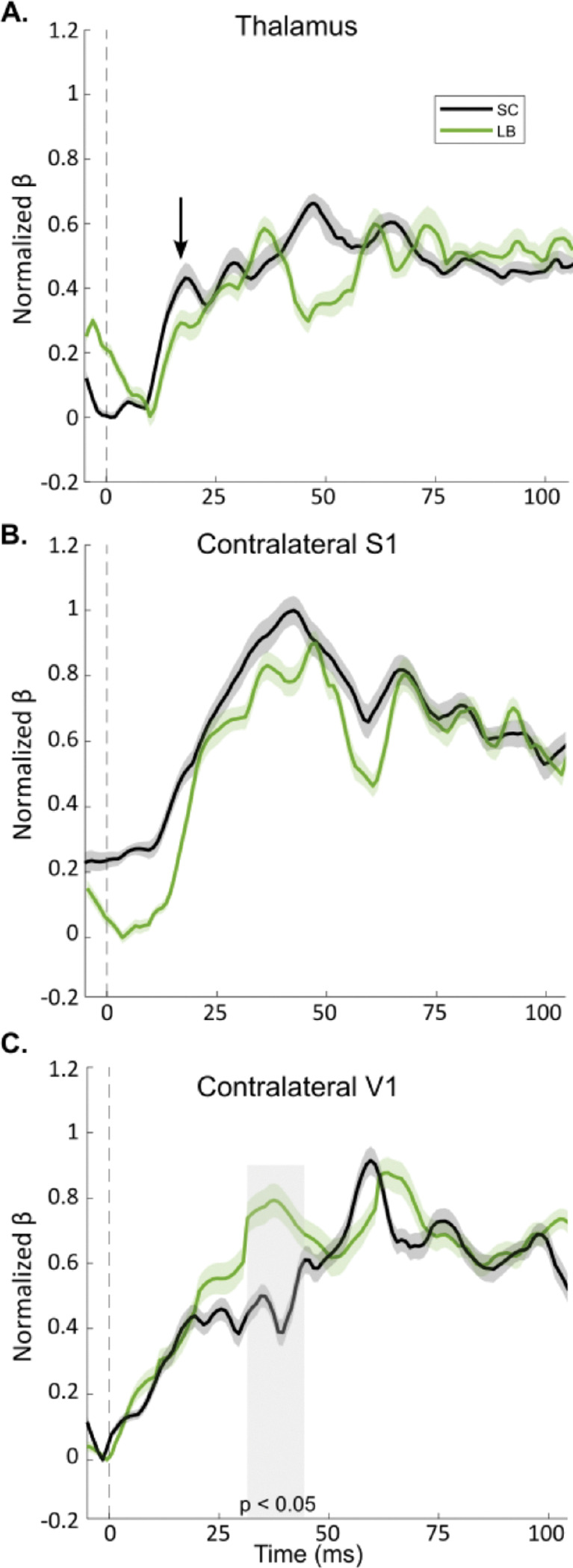
Multiple linear regression model of ROI contribution to the MEG data following tactile stimulation. Data show the coefficients of contribution to tactile stimulation (normalized beta) A. Thalamus. The first peak of contribution is presented by the black arrow. **B.** Contralateral S1. **C.** Contralateral V1. The onset of the tactile stimulation is highlighted by the dashed vertical bar. Shading indicates the standard error of the mean (SEM) for each group. Gray-shaded boxes highlight segments of significant group differences (p < 0.05). LB: late blind individuals; ms: millisecond; SC: sighted controls; S1: primary somatosensory cortex; V1: primary visual cortex.

The earliest somatosensory response in the thalamus emerged around 12 ms post-stimulation in both groups. In LB participants, the first thalamic peak occurred at 17 ms, whereas SC participants showed a slightly delayed and larger peak at 20 ms; these group differences were not statistically significant (*p* = 0.17; Fig. 3A). Following thalamic activation, S1 exhibited rapid increases in beta coefficients starting around 20 ms. In LB participants, the first S1 peak occurred at 34 ms, while in SC participants, the peak occurred at 41 ms. Both groups maintained sustained S1 responses throughout the post-stimulus window, with no significant differences in magnitude (*p* = 0.16; Fig. 3B) or timing (*p* = 0.26; Fig. 3B). The most pronounced group differences were again observed in V1. In that ROI, LB participants showed significantly higher beta coefficients between 29 and 43 ms post-stimulation compared with SC (*p* = 0.04, Bonferroni corrected; Fig. 3C) and exhibited a second V1 contribution at 64 ms. In SC participants, the first significant V1 contribution occurred at 59 ms, slightly earlier than the second peak in the LB group. Overall, these results indicate in both groups a consistent spatiotemporal sequence of neural responses to tactile stimulation. Activation begins in the thalamus (12–20 ms), propagates to S1 (28–49 ms), and subsequently reaches V1. While S1 responses were comparable between LB and SC participants, V1 responses were notably faster and stronger in LB individuals, with an early peak around 35 ms and a second peak at 64 ms. The first peak latencies for all three ROIs are summarised in Table 3.

**Figure 4.**
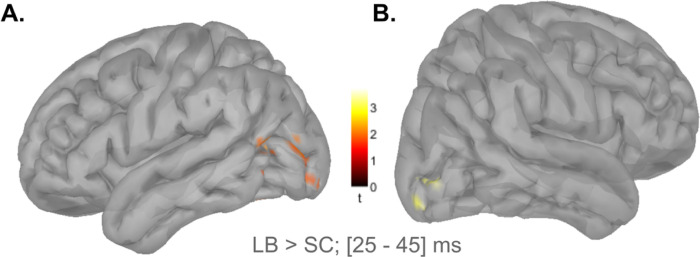
Group differences in cortical responses to tactile stimulation. **A.** Surface projections of the statistical group differences in cortical MEG responses in the contralateral hemisphere following tactile stimulation to the right hand. **B.** Corresponding statistical group differences in cortical responses in the contralateral hemisphere following tactile stimulations to the left hand. LB: late blind individuals; ms: millisecond; SC: sighted controls.

Whole-brain z-score maps closely aligned with the MLR findings. Data were analyzed for the contralateral side following tactile stimulations on the right hand (Fig. 4A) and for the contralateral side following tactile stimulations on the left hand (Fig. 4B). For both sides, LB participants showed superior occipital cortex activity along the calcarine fissure during the 25–45 ms post-stimulation window (Fig. 4A: *p* = 0.02, Bonferroni corrected; Fig. 4B: *p* = 0.01, Bonferroni corrected; permutation test with 5000 randomizations, unequal-variance *t*-test). Such results are consistent with the first V1 peak identified in the MLR model presented in Fig. 3C.

#### Directed functional connectivity

**Figure 5.**
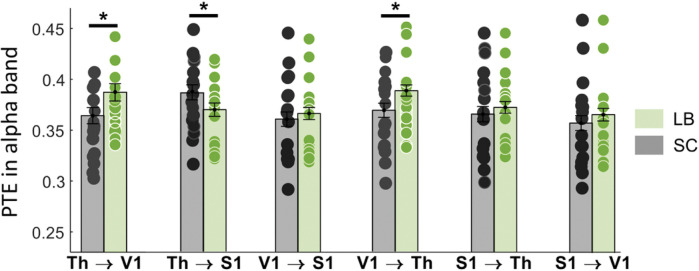
Group differences in directed functional connectivity in response to a tactile stimulus, measured with phase transfer entropy (PTE). PTE measures in the alpha (8–12 Hz) bands are presented for the six possible directions between the thalamus, S1 and V1. Significant results at p < 0.05 are highlighted by an asterisk (*). LB: late blind individuals; SC: sighted controls; S1: primary somatosensory cortex; Th: thalamus; V1: primary visual cortex.

We analyzed directed functional connectivity between the three ROIs from −100 ms to 500 ms relative to the onset of the electro-tactile stimulus. Using PTE, we quantified the communication dynamics among the ROIs in alpha (8–12 Hz), beta (12–30 Hz) and gamma (40–80 Hz) bands. For beta and gamma bands, PTE values were consistent across all six possible ROI interactions, with no group difference being statistically significant. However, significant group differences were observed in the alpha band for three connections. We found increased alpha PTE values in LB compared to SC for bidirectional connectivity between the thalamus and V1 (Fig. 5; Th to V1: *p* = 0.03, Bonferroni corrected; V1 to Th: *p* = 0.04, Bonferroni corrected). In contrast, alpha PTE values were lower in the LB group in the direction from thalamus to S1 (Fig. 5; *p* = 0.03, Bonferroni corrected).

#### Effect of blindness duration

We also assessed the impact of blindness duration, using the BDI scores, on right-hand reaction times, the strength and latency of the ROI responses to tactile stimulation, and PTE values across connections between the three ROIs. This analysis was restricted to the three measures that had shown significant group differences: right-hand reaction time, early V1 responses in LB participants (MLR model), and alpha-band PTE connectivity. There was no statistically significant correlation between BDI and any of the measures tested.

### Auditory processing

#### Imaging Results

**Figure 6.**
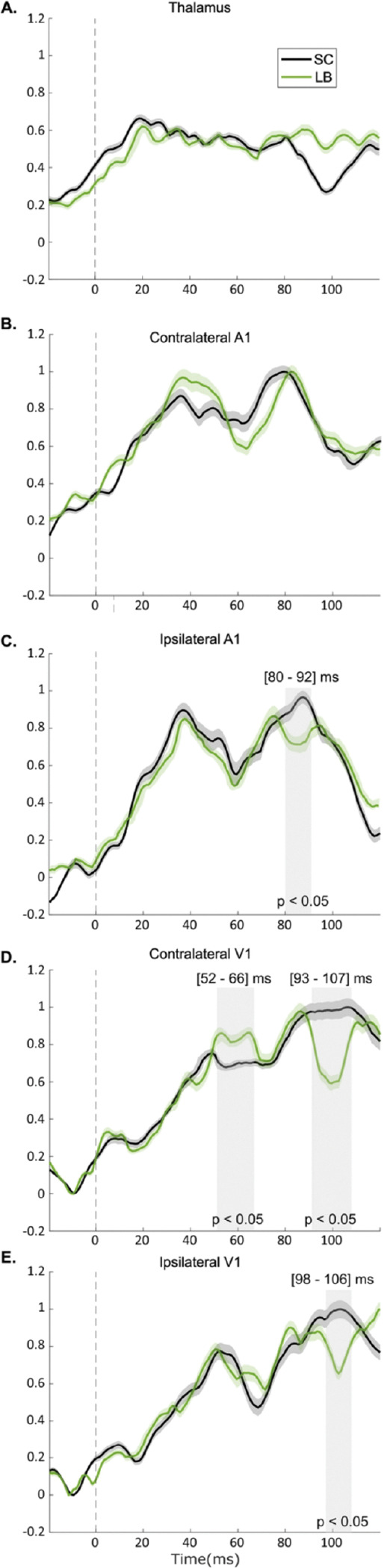
Multiple linear regression model of ROI contribution to the MEG data following auditory stimulation. Data show the coefficients of contribution to the auditory cue (normalized beta) **A**. Thalamus. **B**. Contralateral A1. **C**. Ipsilateral A1. **D**. Contralateral V1. **E**. Ipsilateral V1. The auditory cue occurred at 0 ms and is highlighted by a dashed vertical line. Shading indicates the standard error of the mean (SEM) for each group. Gray-shaded boxes highlight time segments of significant group differences (LB vs SC; p < 0.05). LB: late blind individuals; SC: sighted controls ms: millisecond.

Our findings revealed a consistent, sequential spatiotemporal activation pattern in response to monaural auditory cues across the SC and LB groups. Thalamic activity showed no significant differences between left vs. right ear stimulation or contralateral vs. ipsilateral hemispheres, allowing us to pool the data for greater statistical power of our sample. Using the MLR model (Fig. 6A), early thalamic responses were observed at 19 ms in SC and 20 ms in LB, which did not differ significantly (*p* = 0.87). In A1, activity rose with onset at 20 ms, and the first contralateral contributions recorded at 38 ms in both groups (Fig. 6B), and the first ipsilateral contributions at 36 ms (SC) and 37 ms (LB). A secondary A1 response peaked contralaterally at 82 ms (LB) and 79 ms (SC), and ipsilaterally at 76 ms (LB) and 88 ms (SC). The SC group exhibited significantly stronger ipsilateral A1 activation in the 80–92 ms window (Fig. 6C; *p* = 0.01, Bonferroni corrected). There was a non-significant trend toward stronger contralateral A1 activity in the LB group in the 34–48 ms window (*p* = 0.08). V1 responses followed A1 with a 10–15 ms delay. Contralateral peaks occurred at 53 ms (LB) and 49 ms (SC) (Fig. 6D), while ipsilateral peaks occurred at 51 ms (LB) and 52 ms (SC) (Fig. 6E). LB showed enhanced contralateral V1 responses between 52–66 ms after the auditory cue (*p* = 0.04, Bonferroni corrected). Both groups also exhibited a secondary V1 peak at 86 ms. However, the SC group displayed relatively prolonged V1 activation, leading to significant group differences contralaterally (93–107 ms; *p* = 0.01, Bonferroni corrected) and ipsilaterally (98–106 ms; *p* = 0.03, Bonferroni corrected). Table 4 summarizes the first peak latencies for all three ROIs.

**Figure 7.**
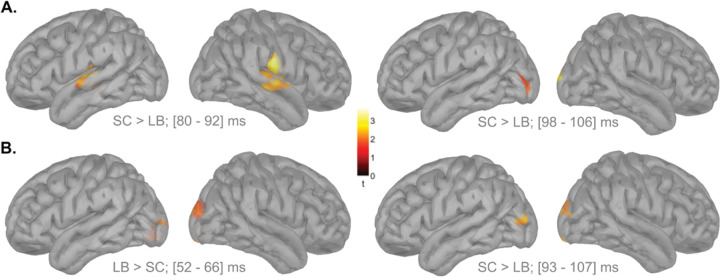
Group differences in cortical responses in response to the auditory cue. **A.**Surface projections of the statistical group differences in cortical MEG responses in the ipsilateral hemisphere following auditory cues. **B.** Corresponding statistical group differences in cortical responses in the contralateral hemisphere following auditory cues. LB: late blind individuals; SC: sighted controls; ms: millisecond.

Since there were no significant differences between left- and right-ear stimulations, data were analyzed by hemisphere (ipsilateral vs. contralateral) and then pooled. Whole-brain z-score maps closely aligned with the MLR findings. In A1, SC participants showed greater ipsilateral activation in the 80–92 ms window (Fig. 7A; *p* = 0.02, Bonferroni corrected, permutation test with 5,000 randomizations, unequal-variance t-test). In the occipital cortex, early responses (52–66 ms post-cue) were stronger in the LB group, consistent with the first V1 peak identified in the MLR model (Fig. 7B; *p* = 0.04, Bonferroni corrected, permutation test with 5,000 randomizations, unequal-variance t-test). In contrast, later occipital responses were stronger in the SC group, with significant differences in both the contralateral (93–107 ms; *p* = 0.03, Bonferroni corrected, permutation test with 5,000 randomizations, unequal-variance t-test) and ipsilateral (98–106 ms; *p* = 0.02, Bonferroni corrected, permutation test with 5,000 randomizations, unequal-variance t-test) hemispheres (Fig. 3B).

### Directed functional connectivity

**Figure 8.**
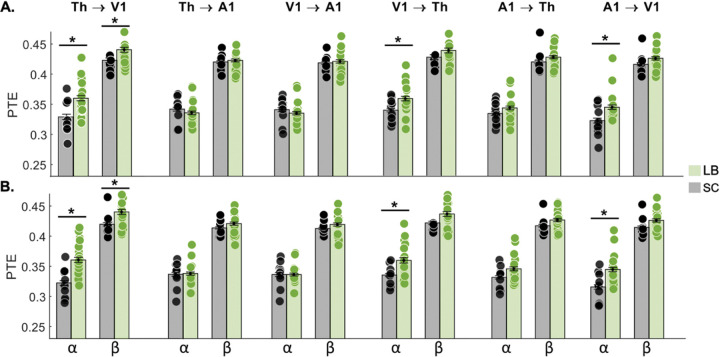
Group differences in directed functional connectivity in response to an auditory stimulus, measured with phase transfer entropy (PTE). PTE measures in alpha (8–12 Hz) and beta (12–30 Hz) bands are presented for the six possible directions between the thalamus, A1 and V1 in both hemispheres. **A.** Results for the contralateral hemisphere of the auditory cue. **B.** Results for the ipsilateral hemisphere of the auditory cue. Significant results at p < 0.05 are highlighted by an asterisk (*). A1: primary auditory cortex; LB: late blind individuals; SC: sighted controls; Th: thalamus; V1: primary visual cortex; a: alpha; b: beta.

PTE analyses revealed significant group differences in directed connectivity across ROIs and frequency bands (Fig. 8A–B). In the alpha band (8–12 Hz), LB participants exhibited higher bidirectional PTE values between the thalamus and V1 in both hemispheres. Similarly, unidirectional alpha connectivity from A1 to V1 was increased in LB in both hemispheres. In the beta band (12–30 Hz), LB participants also showed increased bidirectional connectivity between the thalamus and V1 (all tests for significance: *p* < 0.05, Bonferroni corrected).

### Effect of blindness duration

We then examined whether the BDI and age at blindness onset influenced the normalized beta differences from the MLR model, the Z-score differences in cortical responses, and PTE values across connections between the three ROIs. This analysis was restricted to regions and time windows where significant group differences had been observed. However, there were no significant correlations between either the BDI or age at onset and any of the neural measures tested.

## Discussion

By combining MEG data on spatio-temporal activations and functional connectivity, we were able to trace the neural pathways through which auditory and tactile inputs reach the occipital cortex in LB. A coherent pattern emerges according to a “dual pathway” architecture common to both auditory and tactile modalities, characterized by a transmission process consisting of a fast and a slow pathway working in parallel. However, the anatomical substrates of these pathways differ depending on the sensory modality. For the tactile modality, information is initially transmitted through a rapid thalamo-cortical pathway unique to blind participants, while a subsequent occipital activation arises via a slower, multi-synaptic cortico-cortical route. The fast pathway is functionally consistent with an intra-thalamic reorganization that would allow more direct transmission of tactile information to the occipital cortex via the LGN and optic radiations. For the auditory modality, our results strongly suggest that the rapid transmission of information to V1 occurs via a pre-existing monosynaptic cortico-cortical pathway between A1 and V1, suggesting the use of already established anatomical connections rather than inter-thalamic reorganization A secondary occipital activation is subsequently mediated by a slower, multi-synaptic cortico-cortical pathway.

### Tactile information rerouting to the occipital cortex

The present study provides novel evidence that tactile information is rerouted to V1 in LB individuals through a fast thalamo-cortical pathway. Our findings challenge the traditional notion that such rapid cross-modal rerouting occurs exclusively in cases of early-onset or congenital blindness [28, 29]. Specifically, we observed a distinct sequential responses beginning with a thalamic activation at 17 ms, followed by a S1 response at around 28 ms and a subsequent V1 activation at 34 ms, a sequential timing pattern which fits with the LGN-to-V1 transfer time of 13–19 ms in the human brain [5, 30]. In contrast, the shortest known cortico-cortical rerouting from S1 to V1 involves at least three extra synaptic steps and goes from S1 through the secondary somatosensory cortex (S2), ventral intraparietal area (VIP), tertiary visual cortex (V3), and parieto-occipital area (PO) [14]. The extra synaptic steps each add 5–15 ms, resulting in a total latency of about 60 ms for tactile inputs to reach V1 [14]. This late V1 response (55–70 ms) reported by Müller and colleagues obtained in early blind individuals [5] and present findings in LB align with the estimated polysynaptic response latency.

### Challenging the critical period constraints: thalamo-cortical pathways in late blindness

Animal studies have long demonstrated that early sensory deprivation can lead to profound reorganization of thalamic circuits and that boundaries between thalamic nuclei are not rigid, but are rather malleable under conditions of sensory deprivation [reviewed in 29] [31–34]. Traditionally, such thalamic plasticity has been considered a developmental phenomenon, limited to the early stages of postnatal life. However, a growing body of evidence now challenges this assumption by showing similar morphological alterations in key subcortical structures, irrespective of the age of onset of blindness [reviewed in 28].

For instance, quantitative analyses of the retinofugal projections and the LGN showed no volumetric difference nor in the myelin content between CB and LB participants [35]. A diffusion tensor imaging (DTI) study by Reislev and colleagues [36] identified regional microstructural alterations within the thalamus in both CB and LB individuals, indicating a reorganization of internal thalamic connectivity and a redistribution of fiber orientations between nuclei that may enable residual or novel communication between sensory nuclei, such as between the ventral posterior lateral (VPL) and lateral geniculate (LGN) nuclei [36]. Furthermore, another DTI study, albeit only done in early blind individuals, found a significant reduction in fractional anisotropy (FA) within the optic radiations, indicating decreased white matter integrity, despite the unchanged white matter volume [37]. Although the cortical targets and functional role of these crossing fibers remain unclear, their presence and plasticity indicate a broader capacity for thalamocortical reorganization extending beyond early developmental windows.

Taken together, these results raise the possibility that preexisting but normally silent or weak inter-thalamic connections, or even new connections such as between the VPL and LGN, may either be functionally unmasked or strengthened when sight is lost later in life; our data cannot distinguish between these possibilities. These connections, though limited with normal vision, may become more prominent in blindness through Hebbian mechanisms or homeostatic plasticity [38, 39], thereby enabling tactile information to gain access to visual cortical areas via noncanonical routes.

### Auditory information rerouting to the occipital cortex

The observed timing of neural responses, with thalamic activation at 20 ms, A1 at 38 ms, and V1 at 49–53 ms, is most compatible with auditory inputs reaching the visual cortex via a fast, likely monosynaptic cortico-cortical pathway linking A1 and V1, in line with the 15 ms delay of the responses in A1 and V1. This conjunction is supported by the results of a dynamic causal modeling study using fMRI, which found that reciprocal connections among the MGN, A1, and V1 best explained auditory-driven activity in V1 for both blind and sighted individuals [40]. Consistent with our findings, this model showed increased connectivity strength in blind participants. However, due to the temporal resolution limitations of fMRI, the study could not conclusively distinguish between direct monosynaptic and indirect polysynaptic pathways between A1 and V1. Our MEG results provide complementary temporal precision supporting a direct cortical relay between A1 and V1 and align well with anatomical evidence from non-human primates showing a direct projection from caudal auditory regions including A1, caudal belt, and parabelt areas to V1 regions associated with parafoveal and peripheral visual processing [41, 42].

Following this initial response, a second occipital activation was observed roughly 25–30 ms later. This second activation is likely to reflect a slower, multi-synaptic pathway linking A1 and V1 through the human motion-sensitive complex (hMT+). Situated at the intersection of auditory and visual association cortices, hMT + is well positioned to mediate cross-modal information transfer. It receives convergent input from auditory regions in the superior temporal gyrus and projects back to both early and extrastriate visual cortices [43–45].

Although LB and SC participants exhibited the same overall activation sequence, several differences highlighted unique features of late-onset visual cortex reorganization. LB individuals exhibited enhanced early V1 responses (52–66 ms) but reduced activation for the secondary V1 responses when compared to SC. This pattern contrasts with the sustained and enhanced visual cortex activity typically observed in CB individuals during the performance of auditory tasks [5, 46–51]. This combination of heightened early V1 responses and reduced secondary activations in LB may reflect a form of compensatory excitability plasticity triggered by late blindness [28]. In this case, the amplified initial response may represent the visual cortex’s attempt to boost sensitivity to novel auditory inputs, an adaptation likely driven by rapid, homeostatic adjustments in synaptic strength and excitability within preexisting circuits [52–54]. However, the weaker secondary V1 response in LB and its absence in CB as reported by Paré et al. (under review) [17] suggests that the mature visual cortex can quickly reallocate resources for auditory processing but struggles to sustain prolonged cross-modal engagement. This limitation likely stems from the inherent structural constraints of a system built for vision, which is now forced to handle different sensory inputs without the benefit of developmental reorganization present in CB [28, 55]. Thus, perhaps the mature visual cortex in late blind individuals retains much of its original architectural organization optimized for visual processing, limiting its capacity for extensive cross-modal reorganization compared to the more plastic developing brain [4, 28].

### Frequency specific changes in directed functional connectivity

Our PTE analyses revealed that cross-modal reorganization in LB individuals was characterized by highly frequency-specific changes in directed functional connectivity. Across both analyses, the most consistent effect was enhanced bidirectional connectivity in the alpha-band between the thalamus and V1, closely mirroring prior findings in CB [5]. Alpha oscillations are widely implicated in top-down regulation and predictive signaling [30, 56–60], particularly from deep cortical layers to primary sensory cortices, as part of a mechanism for generating and maintaining top-down predictions [reviewed in 57]. In LB individuals, this enhanced bidirectional alpha-band connectivity between thalamus and V1 may thus reflect the brain’s adaptive integration of tactile and auditory input into repurposed visual circuits through modified thalamocortical feedback loops [5]. These findings are consistent with strengthened communication pathways that facilitate rapid auditory and tactile information transfer to the visual cortex in LB individuals, which is consistent with other findings of thalamocortical reorganization in both early and late blind individuals [5, 36, 37, 61].

Beyond this shared feature, the two modalities exhibited distinct connectivity signatures. In the auditory domain, LB individuals additionally showed beta-band increases in thalamus–V1 coupling, along with enhanced unidirectional alpha-band connectivity from A1 to V1. This is in contrast with recent results from our group showing that increases in CB individuals were solely in the alpha band, with no corresponding beta-band enhancements (Paré et al., under review) [17]. This divergence highlights fundamental differences in the mechanisms of cross-modal plasticity between congenital and late-onset blindness. Furthermore, the absence of any association between these oscillatory increases and either BDI scores or age at blindness onset indicates that the enhanced alpha and beta activity likely arises from the recruitment of pre-existing large-scale networks supporting predictive coding and top-down integration [62, 63], consistent with the view that LB plasticity predominantly leverages mature circuits, rather than relying solely on developmental remodeling [64–66].

In contrast, in the tactile domain, enhanced thalamus–V1 coupling in the alpha band was accompanied by reduced thalamus-S1 connectivity, pointing to a rerouting of somatosensory signals away from canonical feedforward thalamic pathways and toward a more distributed, cross-modal network that includes the occipital cortex [29]. This reconfiguration parallels the dual-route architecture previously described in CB, where early thalamo-cortical inputs shape V1 activation before slower cortico-cortical signals arrive [5]. Table 5 summarises differences in PTE between LB and CB individuals [5, 17].

### How to explain the dichotomy between tactile and auditory processing?

A key question emerging from this study is why intra-thalamic reorganization, which enables rapid rerouting of sensory information, occurs for touch but not for hearing. The difference could be explained by the pre-existing rapid connections between the primary auditory and visual cortices. For hearing, there is already a direct and efficient cortico-cortical pathway between A1 and V1. This monosynaptic connection allows auditory information to reach the occipital cortex rapidly. The gain in processing speed by an additional novel thalamo-cortical rerouting would hence be of little behavioral relevance. Conversely, for the tactile modality, the shortest cortico-cortical pathway between S1 and V1 passes through at least three synapses [5, 28, 29]. In this situation, thalamic reorganization becomes an adaptive solution, bypassing the slower steps via S1 and allowing the tactile signal to reach the occipital cortex quickly. In other words, brain reorganization is a compromise between anatomical constraints and functional needs, which does not entail any reorganization of the thalamus for hearing, since direct exchange between cortical areas already ensures rapid and functional transmission, whereas a tactile-specific inter-thalamic reorganization becomes necessary. Added to this, fast responses to somatosensory stimuli may be of high biological relevance, and important for survival such as in case of exposure to a painful tactile or thermal stimulus. Previous work from our group has for instance shown that pain hypersensitivity in congenital blindness is associated with faster central processing of C-fibre input [67].

### Limited evidence for a graded effect of blindness duration

We did not find any significant correlations between BDI score or age at onset and any of the neural measures showing group differences. Given the relatively small sample size and the scarcity of LB participants with low BDI scores (only two participants had BDI < .50), these null results should be interpreted as indicating limited evidence for a graded relationship in this sample, rather than as evidence for the absence of such a relationship in general. Nevertheless, our findings are consistent with previous reports indicating that functional cross-modal plasticity can occur irrespective of age at onset of blindness onset [68, 69]. This suggests that there is no absolute developmental window for cross-modal plasticity, but that some degree of adaptation can occur rapidly upon onset of vision loss However, the present finding of cross-modal plasticity in LB is in accord with a report that even normally sighted individuals, when blindfolded continuously for just five days, show increased occipital cortex activation during tactile and auditory tasks [70].

## Conclusion

The present study provides electrophysiological evidence that tactile information can reach V1 via two functionally distinct routes: a fast thalamo-cortical pathway observed only in blind individuals, and a slower cortico-cortical pathway that is also present in sighted individuals. In contrast, auditory inputs access the visual cortex of both late-blind and sighted participants through two existing cortico-cortical routes: a rapid monosynaptic projection from A1 to V1, and a slower, polysynaptic pathway. Functional connectivity analyses further revealed strengthened thalamo–V1 coupling in late-blind participants. These findings are largely in line with our previous work in early blindness, but reveal modality- and frequency-specific differences in functional connectivity, particularly in thalamus-S1 and thalamus-V1 coupling, that may distinguish cross-modal reorganization in late- versus early-onset blindness.

## Figures and Tables

**Table 1:** Demographic characteristics of the blind participants.

Participant	Sex	Age	Age onset blindness	BDI	Cause of blindness	Handedness
LB1	M	61	32	0.48	RP	R
LB2	M	56	9	0.84	Retinoschisis	R
LB3	F	43	35	0.19	Fireworks injury	R
LB4	F	57	32	0.44	RP	R
LB5	M	58	41	0.29	RP	R
LB6	M	55	14	0.75	ROP	R
LB7	F	57	7	0.88	ROP	L
LB8	M	56	22	0.61	Chronical Uveitis	R
LB9	M	58	30	0.48	Unknown	R
LB10	M	65	34	0.48	Glaucoma	R
LB11	F	72	27	0.63	Rheumatoid arthritis + iritis	R

BDI: blindness duration index; F: female; L: left-handed; LB: congenitally blind; M: male; R: right-handed; ROP: retinopathy of prematurity; RP: retinitis pigmentosa.

**Table 2:** Number of trials under different experimental conditions.

Normal trials	640 (10 × 64)
Left hand, attended	240 (10 × 24)
Left hand, unattended	80 (10 × 8)
Right hand, attended	240 (10 × 24)
Right hand, unattended	80 (10 × 8)
Cue-only trials	**40 (10 × 4)**
No-trial cue	**40 (10 × 4)**
Total number of trials	**720 (10 × 72)**

**Table 3:** Summary of first peak latencies across our ROIs following tactile stimulations

Group	Thalamus	S1	V1
LB	17 ms	34 ms	35 ms
SC	20 ms	39 ms	59 ms

**Table 4:** Summary of first peak latencies across our ROIs following the auditory cues

Group	Thalamus	A1	V1

LB	20 ms	38 ms - contralateral	53 ms - contralateral
		36 ms - ipsilateral	51 ms - ipsilateral

SC	19 ms	38 ms - contralateral	49 ms - contralateral
		36 ms - ipsilateral	52 ms - ipsilateral

**Table 5:** Summary of PTE differences in LB and CB when compared to SC

Synaptic connections	LB compared to SC	CB compared to SC*
Tactile		
Th → V1	α ↑	α ↑
V1 → Th	α ↑	α ↑
Th → S1	α ↓	α ↑
Auditory		
Th → V1	α ↑, β ↑	α ↑
V1 → Th	α ↑	α ↑
A1 → V1	α ↑	α ↑

* The reported differences for CB are from Muller et al., 2019 and Paré et al., under review.

## Data Availability

The datasets generated and/or analyzed during the current study are available from the corresponding author on reasonable request.
